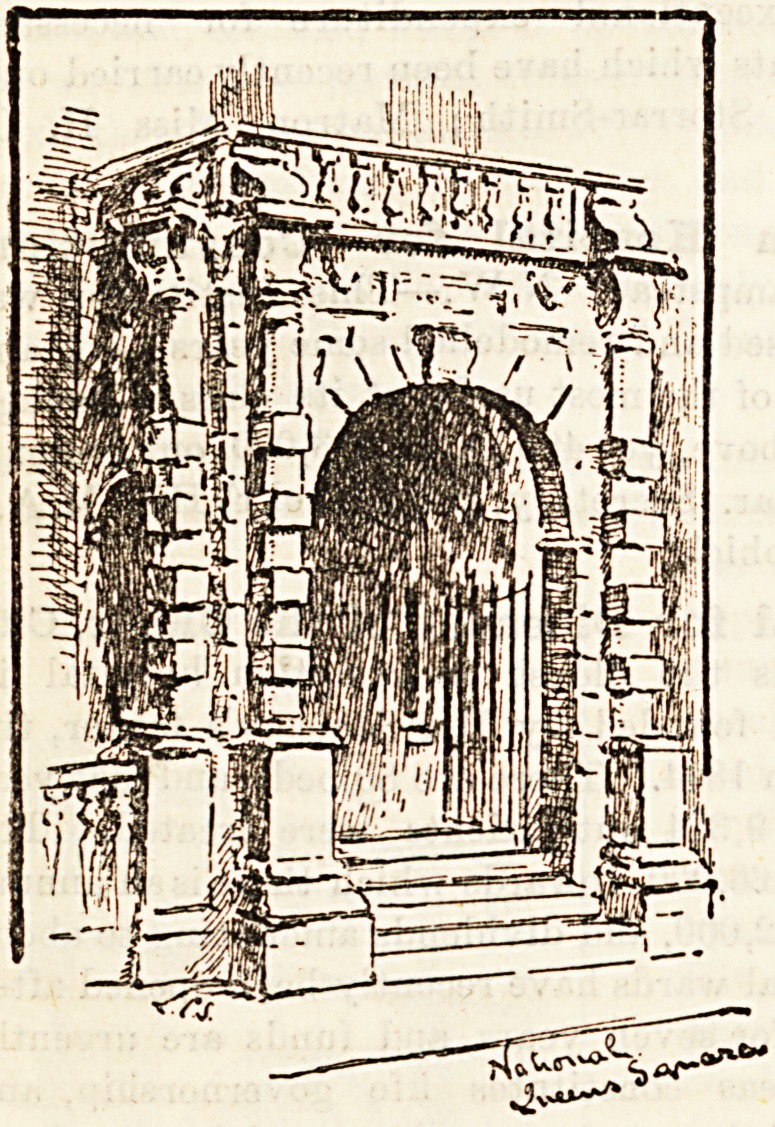# Epilepsy and Paralysis

**Published:** 1893-12-23

**Authors:** 


### EPILEPSY AND PARALYSIS.

**Hospital for Epilepsy and Paralysis and other Diseases of the Nervous System**, Regent's Park, N.W.—At this institution, with the object of promoting providence and minimising abuse, all in and out patients are encouraged to pay what they can afford. Such payments do not, however, nearly cover the cost of maintenance and treatment, while those unable to pay receive free relief. The hospital being unendowed, there necessarily remains a large sum to be collected each year, and the committee, therefore, are appealing for at least £1,000 in new annual subscriptions, and for £1,000 donations to meet the requirements of the coming year. Those who are unable to render more extensive assistance to this hospital might write to the Secretary for copies of his new setting of Lord Tennyson's "Break, Break, Break," dedicated to him by his special permission, and now on sale at 2s. per copy. We have already spoken in warm terms of this composition. Secretary, Mr. Howgrave Graham; Matron, Miss Ridley.

**National Hospital for the Paralysed and Epileptic** (Albany Memorial), Queen Square, W.C.—A class of patients, to whose varied and terrible sufferings no one can deny sympathy, is treated at this hospital. Little provision is made elsewhere for the adequate medical relief of sufferers from nervous diseases, described as the "direst" which afflict humanity, and whose treatment is necessarily protracted beyond the limits possible in a general hospital. There are 180 beds, a number painfully insufficient to meet the requirements virtually of a kingdom, and the attendances of out-patients is about 30,000 yearly. How widespread the work is, may be gathered when it is stated that more than 1,500 different cities, towns, and villages have sent in patients. Besides the hospital for medical and surgical treatment, there is a pension fund for the incurable. Annual expenditure is about £14,000, of which more than £8,000 must be raised in benefactions. Director, Mr. B. Burford Rawlings; Matron, Miss L. C. East.

## Figures and Tables

**Figure f1:**